# Inhibition of alpha-synuclein aggregation by multifunctional dopamine agonists assessed by a novel *in vitro* assay and an *in vivo Drosophila* synucleinopathy model

**DOI:** 10.1038/srep38510

**Published:** 2016-12-05

**Authors:** Deepthi Yedlapudi, Gnanada S. Joshi, Dan Luo, Sokol V. Todi, Aloke K. Dutta

**Affiliations:** 1 Department of Pharmaceutical Sciences, Wayne State University, Detroit, MI 48202,USA; 2 Department of Pharmacology, Wayne State University, Detroit, MI 48201, USA.

## Abstract

Aggregation of alpha synuclein (α-syn) leading to dopaminergic neuronal death has been recognized as one of the main pathogenic factors in the initiation and progression of Parkinson’s disease (PD). Consequently, α-syn has been targeted for the development of therapeutics for PD. We have developed a novel assay to screen compounds with α-syn modulating properties by mimicking recent findings from *in vivo* animal studies involving intrastriatal administration of pre-formed fibrils in mice, resulting in increased α-syn pathology accompanying the formation of Lewy-body (LB) type inclusions. We found that *in vitro* generated α-syn pre-formed fibrils induce seeding of α-syn monomers to produce aggregates in a dose-and time-dependent manner under static conditions *in vitro*. These aggregates were toxic towards rat pheochromocytoma cells (PC12). Our novel multifunctional dopamine agonists D-519 and D-520 exhibited significant neuroprotection in this assay, while their parent molecules did not. The neuroprotective properties of our compounds were further evaluated in a *Drosophila* model of synucleinopathy. Both of our compounds showed protective properties in fly eyes against the toxicity caused by α-syn. Thus, our *in vitro* results on modulation of aggregation and toxicity of α-syn by our novel assay were further validated with the *in vivo* experiments.

Parkinson’s disease (PD) is the second most common neurodegenerative disorder after Alzheimer’s disease, afflicting nearly 1% of the population over the age of 60 years. PD is pathologically characterised by the dopaminergic neuronal loss in the substantia nigra pars compacta and other regions of the brain resulting in the degeneration of the nigro-striatal tract and loss of dopamine (DA)^1^ and by the presence of Lewy bodies and Lewy neurites consisting primarily of α-synuclein (α-syn) along with Parkin and ubiquitin^2^. α-Syn is a small, 14-kDa, acidic, natively unfolded protein^3^, which has been linked to vesicular trafficking, neurotransmitter release, and regulation of neurotransmission^4^. Evidence from human genetics, animal models, and biochemical and biophysical studies suggests that α-syn aggregation plays a central role in the initiation and/or progression of PD^5^. Structurally, α-syn can be divided into three regions with distinct structural characteristics^6^. The N- terminal domain encodes for a series of 11 amino acid imperfect repeats with a consensus motif of KTKEGV, which is capable of forming amphipathic helices^7^. The central hydrophobic region, also known as the non-amyloid beta component is associated with an increased fibril forming potential^8^. The acidic C- terminus domain mainly consists of negatively charged residues and is largely unfolded (Fig. 1B).

Current therapeutic strategies for treating PD mainly offer symptomatic relief by restoring the levels of DA by the administration of levodopa (L-DOPA), a direct precursor of the neurotransmitter. This is used in combination with other drugs like Catechol *O*-methyl transferase inhibitors (COMTI), monoamine oxidase B inhibitors (MAOBI), and peripheral aromatic L-amino acid decarboxylase inhibitors (AADCI) that help in slowing down the degradation of L-DOPA in the periphery and increasing its availability. However, long term use of L-DOPA gives rise to motor fluctuations with dyskinesias and a decrease in the duration of response time to a given L-DOPA dose^9^. Moreover, prolonged use of L-DOPA gives rise to “on” and “off” episodes and might lead to toxicity to DA neurons, hence accelerating the DA neurodegeneration process^10^,^11^. It has been observed that both L-DOPA and DA generate free radicals during normal metabolism^12^ and hence the use of dopamine agonists such as Bromocriptine, Pergolide, Ropinirole, and Pramipexole^13^,^14^,^15^,^16^ have gained a lot of importance for the treatment of PD. However, there is a significant unmet need in the development of symptomatic and disease modifying agents for the treatment of PD.

Due to a lack of thorough understanding of the mechanisms mediating neurodegeneration in PD, the development of therapeutic strategies to block cell death has been limited. However, increasing evidence of a pivotal role of α-syn in the pathogenesis of PD has made it a potential therapeutic target for the development of neuroprotective drugs. The main strategies include the development of anti aggregation compounds, gene silencing approaches and α-syn aggregate clearance strategies^17^. Out of these, inhibition of α-syn aggregation remains an extremely attractive target from drug development perspective. Some of the inhibitors developed include epigallocatechin-3-gallate (EGCG)^18^, 3-(1,3-benzodioxol-5-yl)-5-(3-bromophenyl)-1*H*-pyrazole (anle138b)^19^, CLR01^20^ and the prolyl oligopeptidase inhibitor KYP-204798^21^.

Recombinant α-syn can form filamentous aggregates *in vitro* similar to those found in the human brain^22^,^23^,^24^,^25^. However, it has been observed that under static conditions, the generation of fibrils takes several days and the concentration of recombinant protein needed is notably high, between 300 to 500 μM^26^,^27^. In order to accelerate this process, several factors have been utilized. These include the use of anions^28^, polyanions^29^,^30^, polycations^31^, salts^28^, pesticides and herbicides^32^,^33^,^34^, heavy metals^35^, negatively charged detergents^36^, molecular crowding^37^,^38^,^39^, low pH^40^ and acidic phospholipids^41^,^42^. Apart from these factors, agitation has been shown to induce aggregation and several assays have been developed based on this property to screen α-syn aggregation inhibitors^43^.

Due to the complex pathogenesis of PD, it is recognized that a multifunctional drug addressing the underlying disease processes will be more effective than a drug targeting a single pathological feature of the disease. In our approach, along this line, we designed compounds with both symptomatic and neuroprotective disease modifying effects. Thus, we have designed and biologically characterized multifunctional dopamine agonists D-519 and D-520 as modulators of α-syn aggregation^44^.

As described above, all the methods for *in vitro* aggregation of α-syn are intensely aggressive and artificial in the context of its aggregation in the PD brain. In order to overcome this drawback, we have developed a novel assay for the screening of α-syn aggregation inhibitors, wherein monomeric α-syn is seeded with pre-formed fibrils (PFFs) without agitation. It has been shown that α-syn fibrils can seed Lewy body and Lewy neurite like inclusions in the cell culture models^22^,^23^. Inoculation of PFFs into mice brain seeds aggregation of the endogenous α-syn and shows neurodegeneration similar to PD^24^. Similar results have been reported in a rat model recently^45^. Our novel method was informed by these recent observations.

Our assay, apart from mimicking the conditions that are likely occurring in the PD brain, drastically reduces the time as well as the concentration of α-syn required for aggregation. We observe the formation of aggregates in a time dependent manner. The progression in aggregate formation has been studied by Thioflavin T (ThT) assay, transmission electron microscopy (TEM) and circular dichroism (CD) spectroscopy. We show here that multifunctional dopamine agonists, D-519 and D-520 (Fig. 1A), developed by us are capable of inhibiting α-syn aggregate formation, which resulted in reduction of toxicity. Furthermore, the protective effects of the compounds observed in our novel assay were further confirmed and validated in a GFP-based *Drosophila* model expressing mutant pathogenic A30P α-syn protein in fly eyes to produce detectable and quantifiable toxicity.

## Results

### Effect of seeding on the aggregation of monomeric α-syn

The formation of PFFs was confirmed by ThT assay. It was observed that there was nearly a 200 fold increase in the ThT value when compared to monomeric α-syn (Supplementary Fig. 1). To assess the ability of PFFs to induce aggregation of monomeric α-syn, the PFFs at 1% v/v were incubated with 1.25 mg/mL α-syn at 37 °C without agitation for a period of 10 days (D). The degree of fibrillation was measured by ThT assay. It was observed that there was nearly a 20 fold increase in ThT fluorescence value when compared to monomeric α-syn at 0D (Supplementary Fig. 2). However, we wanted the aggregation to take place more gradually in order to mimic the PD conditions. We therefore reduced the concentration of PFFs used for seeding to 0.5% and also increased the incubation time to 30D.

### Effect of 0.5% PFFs on the aggregation of monomeric α-syn

**Figure 2:**
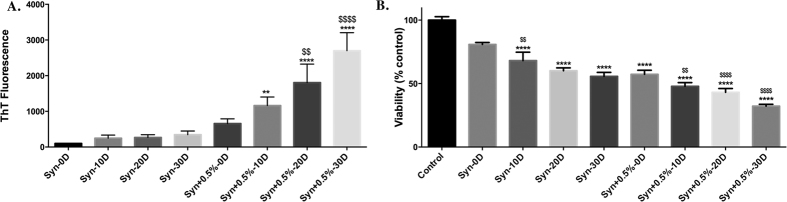
Effect of 0.5% PFF on the aggregation of monomeric α-syn. 1.25 mg/mL α-syn was incubated with 0.5% PFFs for a period of 30D. (**A**) Fibrillation was measured by ThT assay. Values are represented in terms of % 0D Synuclein. (**B**) Effect of the aggregates formed by 0.5% seeding on the viability of PC12 cells; PC12 cells were treated with 10 μM aggregates formed for a period of 24 H, following which cell viability was measured by MTT assay. Values are represented in terms of % control. Data values shown are means ± SD of three independent experiments. One-way ANOVA analysis followed by Tukey’s multiple comparison post hoc test was performed, **p ≤ 0.01, ****p ≤ 0.0001 compared to Syn-0D; ^$$^*p* ≤ 0.01, ^$$$$^*p* ≤ 0.0001 compared to Syn + 0.5%-0D.

Upon incubation of monomeric α-syn with 0.5% PFFs, it was observed that there was a time dependent increase in the aggregation of α-syn as evidenced by ThT fluorescence. There was nearly a 11.6, 18 and 27 fold increase in the ThT value at 10D, 20D and 30D, respectively when compared to monomeric α-syn at 0D (Fig. 2A). Thus, the rate of formation of aggregates as indicated by ThT fluorescence was slower compared to the aggregates formed by 1% PFF seeding at 10D (Supplementary Fig. 2). In order to examine the cytotoxicity of the aggregates formed towards PC12 cells, α-syn aggregates at a concentration of 10 μM were incubated with the cells for a period of 24 H, following which cell viability was measured by MTT assay. It was observed that the aggregates generated from incubation for 30D seeding were most toxic when compared to the corresponding 10D and 20D α-syn seeding samples. The decrease in viability caused by the 0D, 10D, 20D and 30D seeded samples was 23%, 32%, 37% and 48% respectively when compared to the cells treated with monomeric α-syn (Fig. 2B). With this result, we decided to carry out our experiments with 0.5% seeding.

### Effect of Rifampicin and the parent compounds 5-OHDPAT and Pramipexole on the aggregation of α-syn with 0.5% seeding

**Figure 3:**
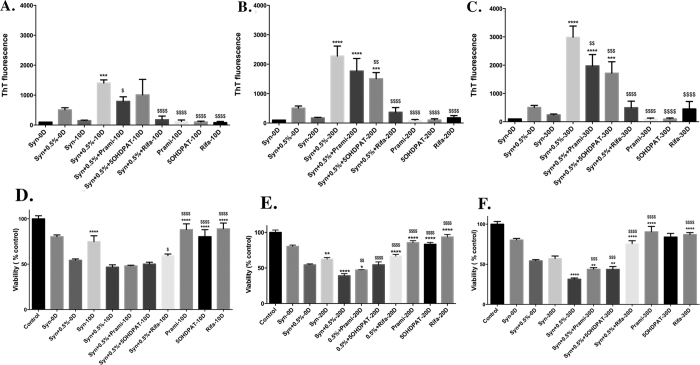
Effect of reference compound and parent compounds on the aggregation of α-syn induced by seeding with 0.5% PFFs. 1.25 mg/mL α-syn was incubated with 0.5% PFFs for a period of 30D without shaking in the presence of Rifampicin or Pramipexole or 5-OHDPAT at a concentration of 172.9 μM. Fibrillation was measured by ThT assay at 10D (**A**), 20D (**B**), 30D (**C**). Values are represented in terms of % 0D Synuclein. Viability of PC12 cells was measured by MTT assay after 24 h treatment with α-syn seeded samples collected at 10D (**D**), 20D (**E**), 30D (**F**). Values are represented in terms of % control. Data values shown are means ± SD of three independent experiments. One-way ANOVA analysis followed by Tukey’s multiple comparison post hoc test was performed, *p ≤ 0.05, **p ≤ 0.01, ***p ≤ 0.001, ****p ≤ 0.0001 compared to Syn + 0.5%-0D; ^$^*p* ≤ 0.05, ^$$^*p* ≤ 0.01, ^$$$^*p* ≤ 0.001, ^$$$$^*p* ≤ 0.0001 compared to Syn + 0.5%–10D (**A,D**) or Syn + 0.5%-20D (**B,E**) or Syn + 0.5%-30D (**C,F**).

It has been shown that Rifampicin inhibits α-syn fibrillation and disaggregation of fibrils via preferential stabilization of monomeric and soluble oligomeric forms^46^. α-Syn seeded with 0.5% PFFs was incubated with twice the molar concentration (172.9 μM) of either Rifampicin (reference compound) or the parent compounds Pramipexole or 5-OHDPAT for a period of 30 days. Figure 3A,B,C represent ThT values at 10D, 20D and 30D, respectively. Out of the three compounds, Rifampicin showed a pronounced effect on the inhibition of α-syn aggregation right from 10D until 30D. The decrease in the ThT fluorescence shown by Rifampicin, Pramipexole and 5-OHDPAT at 30D was by 6, 1.5 and 1.7 fold respectively when compared to 30D seeded α-syn (Fig. 3C). The aggregates formed in the presence of these compounds were treated to PC12 cells. Figure 3D,E and F represent the viability of PC12 cells when treated with α-syn aggregates collected at 10D, 20D and 30D respectively. It was observed that the aggregates formed in the presence of Rifampicin, Pramipexole and 5OHDPAT were less toxic when compared to seeded α-syn. The increase in the viability was 44%, 12% and 12% respectively when compared to α-syn seeded with 0.5% PFFs alone at 30D (Fig. 3F).

### Effect of multifunctional dopamine D_2_/D_3_ agonists D-519 and D-520 on inhibition of aggregation of α-syn

**Figure 4:**
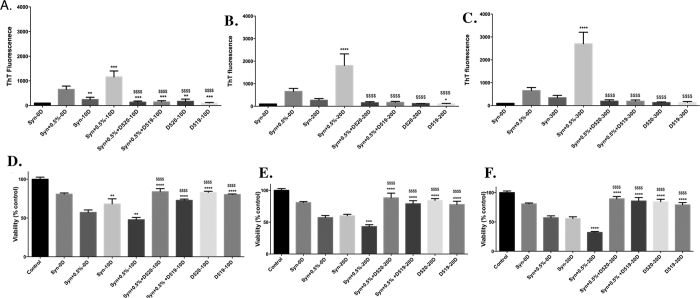
Effect of D-520 and D-519 on the aggregation of α-syn induced by seeding with 0.5% PFFs. 1.25 mg/mL α-syn was incubated with 0.5% PFFs for a period of 30D without shaking in the presence of D-520 or D-519 at a concentration of 172.9 μM. Fibrillation was measured by ThT assay at 10D (**A**), 20D (**B**), 30D (**C**). Values are represented in terms of % 0D Synuclein. Viability of PC12 cells was measured by MTT assay after 24 h treatment with α-syn seeded samples collected at 10D (**D**), 20D (**E**) and 30D (**F**). Values are represented in terms of % control. Data values shown are means ± SD of three independent experiments. One-way ANOVA analysis followed by Tukey’s multiple comparison post hoc test was performed, *p ≤ 0.05, **p ≤ 0.01, ***p ≤ 0.001, ****p ≤ 0.0001 compared to Syn + 0.5%-0D; ^$$$$$^*p* ≤ 0.0001 compared to Syn + 0.5%-10D (**A,D**) or Syn + 0.5%-20D (**B,E**) or Syn + 0.5%-30D (**C,F**).

The dopamine D_2_/D_3_ agonist compounds D-520 and D-519, which have been synthesized by us, were incubated with α-syn seeded with 0.5% PFFs at twice the molar concentration (172.9 μM) of α-syn for a period of 30D at 37 °C without agitation. Both D-520 and D-519 lead to a significant decrease in the ThT fluorescence (p < 0.0001) when compared to α-syn seeded with 0.5% PFFs alone. This effect was consistent until 30D (Fig. 4A,B,C). There was a 1.7, 2.7 and 4.1 fold increase in the ThT value of the seeded samples at 10D, 20D and 30D respectively, when compared to 0D seeded sample (Fig. 2A). In the presence of our compounds, D-520 and D-519, the ThT value was reduced almost close to the value shown by α-syn alone at 0D, showing that our compounds inhibited α-syn aggregation very efficiently and this effect was consistent until 30D.

When PC12 cells were treated with the aggregates formed by 0.5% PFFs seeding, it was observed that the viability went down to 47%, 43% and 32% for 10D, 20D and 30D samples, respectively, compared to control (Fig. 2B). However, the seeded samples formed in the presence of D-520 and D-519 showed less toxicity when compared to their respective time point’s seeded samples without compounds. The viability values of PC12 cells when treated with α-syn seeded samples in the presence of D-520 and D-519 were 84% and 73% at 10D; 88% and 79% at 20D and 90% and 86% at 30D respectively when compared to control (Fig. 4C,D,E).

### Morphology of aggregates by Transmission Electron Microscopy

**Figure 5:**
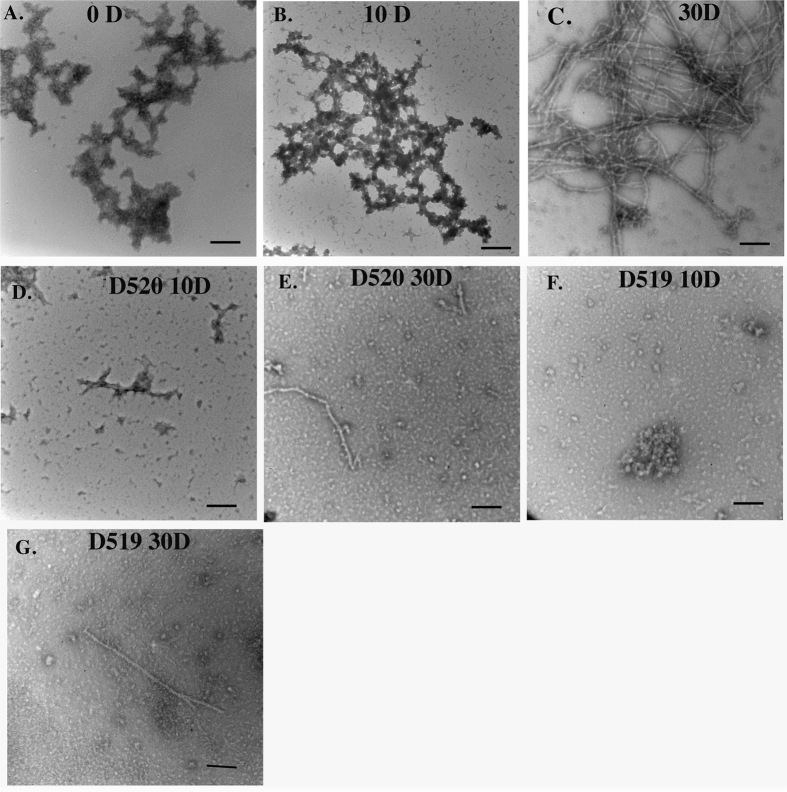
TEM analysis of α-syn seeded with 0.5% PFF. (**A**) at day 0, (**B**) at day 10, (**C**) at day 30, (**D**) In the presence of D-520 at day 10, (**E**) In the presence of D-520 at day 30, (**F**) In the presence of D-519 at day 10, (**G**) In the presence of D-519 at day 30; Scale bar = 100 nm.

The results from ThT and cytotoxicity data were further corroborated by Transmission Electron Microscopy (TEM). There was a gradual increase in the amount of aggregates formed in the seeded samples. The 0D sample of α-syn seeded with 0.5% PFFs showed large fibrillar structures recruiting the monomers of α-syn. By the end of 30D, these transformed into fibrillar structures which are highly intertwined, complementing the ThT data (Fig. 5A,B,C). In contrast, the compounds D-520 and D-519, lead to the formation of small amorphous aggregates with a few detectable fibrils (Fig. 5D,E,F,G).

### Secondary structure changes of α-syn monitored by far UV CD

**Figure 6:**
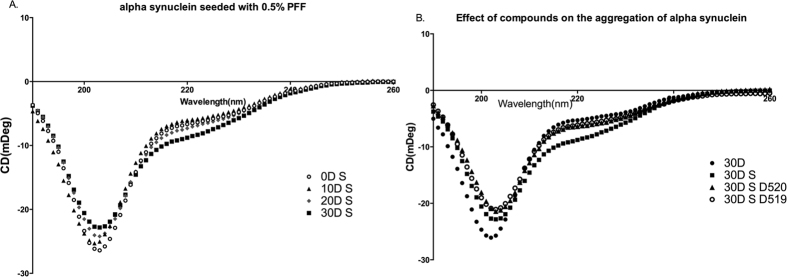
Secondary structure analysis of the aggregates. Circular dichroism spectra analysis of (**A**) the seeding samples at different time points (**B**) aggregates formed without or with seeding in the presence of compounds at 30D.

Figure 6A represents the far-UV CD spectra of α-syn seeded with 0.5% PFFs at various time points. The CD changes are a function of incubation time from 0D to 30D. There is an increase in the intensity of the negative shoulder at about 220 nm and a decrease in the intensity of the negative shoulder at about 203 nm. These observations are consistent with the data showing that the degree of aggregation increases with incubation time^47^.

In Fig. 6B, the CD spectra of 30D samples have been plotted. It was observed that 30D seeding sample shows an increase in the intensity of the negative shoulder at about 220 nm and a decrease in the intensity of the negative band at about 203 nm representative of a more structured form, similar to the β-peptide backbone structure, when compared to α-syn alone. The presence of compounds D-520 and D-519 showed a somewhat different trend which involved similar absorption behavior to unseeded α-syn at 220 nm but decrease in absorption at 203 nm compared to unseeded and seeded α-syn samples alone.

### α-Syn dependent toxicity in *Drosophila*

**Figure 7:**
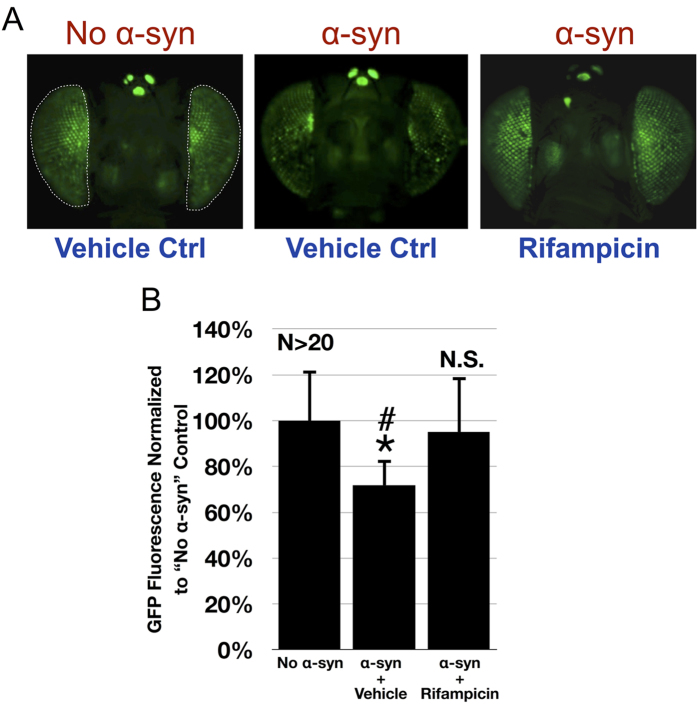
A *Drosophila* model of α-syn toxicity and the protective effect from Rifampicin. (**A**) Representative images of fly eyes not expressing or expressing α-syn^A30P^ through the Gal4-UAS system. Driver was GMR-Gal4, which expresses UAS-α-syn in fly eyes. All flies also expressed membrane-targeted GFP (mCD8-GFP), which is independent of the α-syn transgene. Flies were heterozygous for all transgenes. Adult flies were placed in food that contained Rifampicin (1 mg/mL in DMSO) or the vehicle control (DMSO alone) as soon as they emerged from the pupal case and were fed for 28 days, with food changed every three days. Images are from flies that were 28 days old. Dotted lines: Areas quantified in panel B. (**B**) Quantification of fly eyes from panel A and other flies with the same genotypes. Average GFP fluorescence was normalized to fluorescence from no α-syn, vehicle-treated control flies. *p < 0.05 comparing vehicle treated α-syn eyes to no α-syn column, ^#^*p* < 0.05 comparing vehicle treated α-syn eyes to Rifampicin treated α-syn eyes, N.S.: statistically not significant, comparing no α-syn eyes to Rifampicin treated α-syn eyes. Values are represented as means ± SD. Statistics: one way ANOVA with Tukey’s post-hoc.

**Figure 8:**
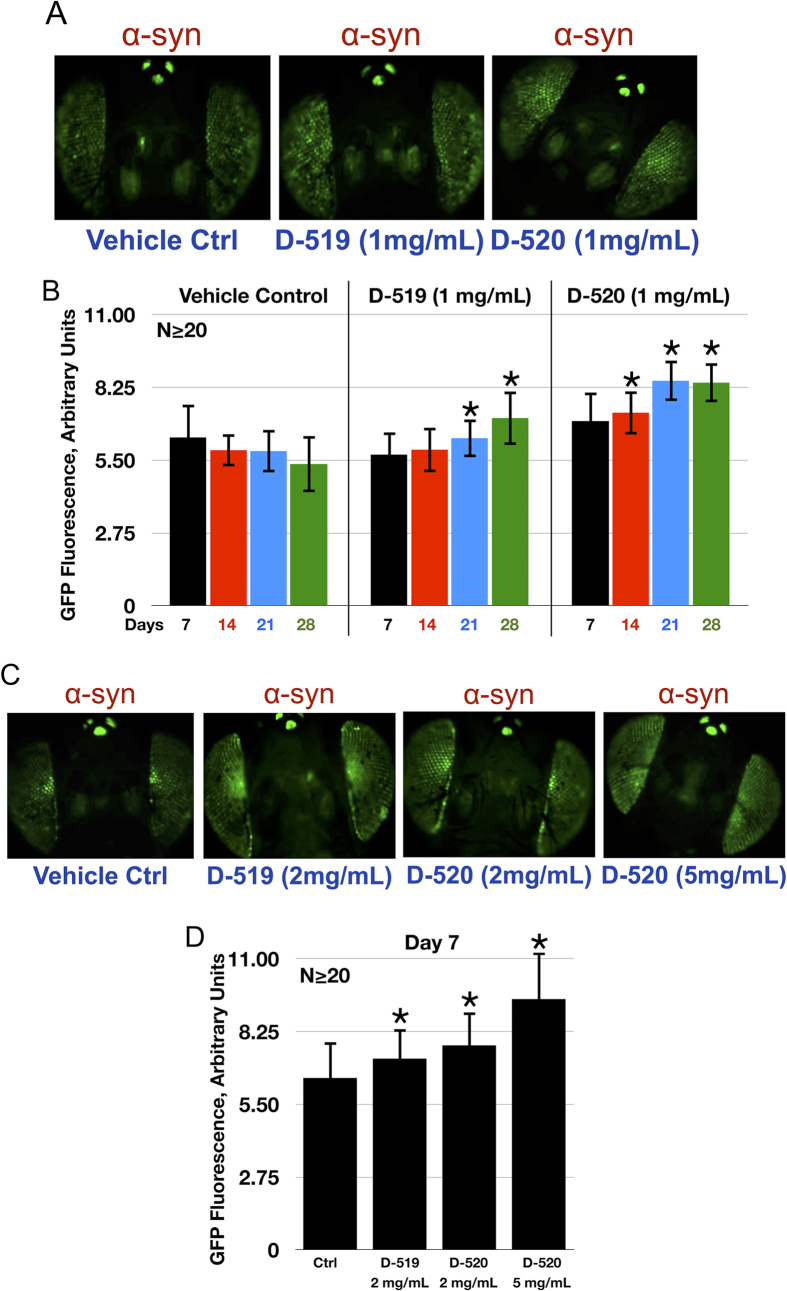
Protective effect from D-519 and D-520 in fly eyes expressing α-syn. (**A**) Representative images of flies expressing α-syn^A30P^ through the Gal4-UAS system. Flies were fed the vehicle control (ultra pure water) or D-519 or D-520 (1 mg/mL in ultra-pure water) for 28 days. Flies were heterozygous for driver (GMR-Gal4), α-syn^A30P^and mCD8-GFP. (**B**) Quantification from fly heads shown in panel A and other flies that were fed vehicle control (ultra-pure water), D-519 or D-520 for the indicated amounts of time. *p < 0.05 based on one way ANOVA with Tukey’s post-hoc comparing flies fed the compound to same-day flies fed with vehicle control. (**C**) Representative images of fly eyes expressing α-syn and which were fed with either the vehicle control or the indicated compounds at different concentrations dissolved in ultra-pure water. (**D**) Quantification of eye fluorescence from α-syn-expressing flies that were fed vehicle control, D-519 or D-520 at the indicated concentration for 7 days after the adults emerged from their pupal cases. *p < 0.05 based on one way ANOVA with Tukey’s post-hoc correction comparing compound-fed flies to “ctrl” ones that were fed the vehicle control. Values are represented as means ± SD.

We examined the efficacy of compounds D-519 and D-520 against α-syn-dependent toxicity in a model of synucleinopathy in the fruit fly *Drosophila melanogaster* by utilizing a highly sensitive and quantitative method which we described recently^48^. Briefly, this technique relies on a version of GFP that is membrane bound. As eye cells degenerate, GFP fluorescence is lost and can be quantified when imaged through a fluorescent microscope (Figs 7 and 8). The GFP reporter faithfully tracks internal eye degeneration and the technique can be used to identify modifiers of various neurotoxic proteins^48^,^49^.

As a proof of principle for the ability of our system to pick up molecular suppressors of α-syn-dependent toxicity, we fed flies Rifampicin (1 mg/mL), a known α-syn aggregation inhibitor. As shown in Fig. 7A (quantified in Fig. 7B), when adult flies are fed Rifampicin for a period of 28 days, fluorescence from α-syn expressing flies is similar to that of eyes which do not express α-syn and are fed with the vehicle alone.

Next, we tested whether the flies fed with D-519 or D-520 (1 mg/mL) for a period of up to 28 days would also display a protective effect. As shown in Fig. 8A and B, over the course of time fluorescence decreases from fly eyes that express α-syn and are not fed with either of the compounds. Both D-519 and D-520 are able to suppress this loss of fluorescence and, over time, even reverse it, indicating a strong protective effect against α-syn-induced toxicity in this *in vivo* model. When we examined by native gels the type of α-syn species present in this fly model, we found that, concomitant with the increase in GFP intensity in drug-treated flies, there was a decrease in α-syn aggregated species compared to vehicle treated flies (Supplementary Fig. 3).

Treatment with either D-519 or D-520 led to a stronger suppression of phenotype than Rifampicin at 28 days. From analyzing the extent to which treatment by any of these compounds increased GFP fluorescence in fly eyes that express α-syn, Rifampicin led to an increase of ~30% in GFP intensity (Fig. 7B), whereas D-519 and D-520 led to an increase of ~34% and ~57%, respectively (Fig. 8B), in GFP intensity. Based on these results, these compounds are stronger suppressors of toxicity than Rifampicin in *Drosophila*, which is in agreement with our results from *in vitro* assays (Figs 3 and 4).

The protective effect of both compounds is dose-dependent, as shown in Fig. 8C and D; higher doses lead to stronger suppression, as early as day 7. Collectively, these results indicate that both D-519 and D-520 are protective against α-syn-dependent toxicity in the fly.

## Discussion

α-Syn aggregates have been found in healthy grafted neurons in PD patients and endogenous α-syn has been shown to be recruited into fibrillar aggregates through a seeding mechanism in transgenic mice^24^,^50^. These reports laid the foundation for the importance of seeding or nucleation in the aggregation of α-syn. Several *in vitro* assays for the screening of α-syn aggregation inhibitors have been reported. However, all of these assays are performed under forceful conditions like high speed agitation or under high temperatures, not reflective of actual processes occurring in the human brain. In order to mimic the conditions occurring in the PD brain, here, we have developed a novel *in vitro* assay for the screening of small molecules which inhibit α-syn aggregation. We applied this assay to assess the activity of our novel multifunctional dopamine agonists D-519 and D-520. The utility of this technique was then validated in an *in vivo Drosophila* model.

The method which we have developed is without agitation, under “static conditions”. In order to hasten the aggregation, monomeric α-syn is incubated with PFFs which act as seeds to start the nucleation process. To delineate the concentration of PFFs to be used for seeding of monomeric α-syn, we first seeded α-syn with 1% PFFs at 37 °C without agitation for a period of 10D. Seeding with 1% PFFs lead to a 20 fold increase in the ThT value at the end of 10D when compared to monomeric α-syn at 0D (Supplementary Fig. 2). However, we wanted the aggregation to take place more gradually at a slower pace. Hence we decreased the concentration of PFFs used for seeding to 0.5% and also increased the incubation time to 30D. We observed that 0.5% PFFs lead to an increase in the fibrillation by 11.6, 18 and 27 fold at 10D, 20D and 30D, respectively when compared to monomeric α-syn at 0D. However, the sample without seeding showed only a 3 fold increase in fibrillation (Fig. 2A). Our results are supported by previous findings which demonstrated that, the aggregation of α-syn *in vitro* proceeds in a nucleation dependent process, i.e. fibril formation displays a lag phase followed by an increase in the rate of fibril formation^40^,^51^,^52^. This lag phase has been shown to be reduced significantly by the addition of a seed or a nucleus of pre-aggregated α-syn^51^,^53^.

The aggregates formed by seeding at 30D reduced the viability of PC12 cells by 68% as opposed to 44% by the sample generated without seeding (Fig. 2B), strongly indicating pronounced effect of seeding or nucleation dependent effect on aggregation and toxicity (Fig. 2A,B). The reference compound Rifampicin lead to a decrease in the aggregation of seeded α-syn, as measured by ThT fluorescence (Fig. 3A–C) and was also able to decrease the toxicity of the aggregates formed in its presence towards PC12 cells (Fig. 3D–F).

The multifunctional hybrid compounds D-519 and D-520, were derived from parent precursor molecules Pramipexole and 5-OHDPAT by a fragment based drug development approach^44^,^54^. The rationale behind testing the parent precursor compounds was to evaluate whether these compounds exhibit an inherent modulation of α-syn aggregation property. Our results indicate that the parent compounds, Pramipexole and 5-OHDPAT, were far less effective in inhibiting aggregation and reducing toxicity of α-syn compared to the reference drug Rifampicin (Fig. 3A–F).

We have previously shown that D-520, a multifunctional dopamine agonist developed in our laboratory is a potent α-syn aggregation inhibitor^44^. We therefore tested this compound along with its analog D-519 using the assay developed by us. We observed that compared to the ThT activity of seeded α-syn at 30D, D-520 and D-519 induced a 14 fold decrease in ThT activity (Fig. 4C) whereas the reference compound Rifampicin was able to decrease the ThT activity by 6 fold at 30D (Fig. 3C). Also the parent precursor compounds Pramipexole and 5-OHDPAT showed only a 1.5 and 1.7 fold decrease in ThT value, respectively when compared to the seeded α-syn at 30D (Fig. 3C). α-Syn aggregates formed in the presence of D-520 and D-519 were much less toxic towards PC12 cells. The increase in the viabilities were 57% and 53% respectively when compared to the seeded α-syn formed in the absence of the compounds at 30D (Fig. 4D,E,F). This suggests that multifunctional dopamine agonists D-519 and D-520 show a prolonged activity and are efficient even until 30D. In contrast, Rifampicin, Pramipexole and 5-OHDPAT were able to increase the viability by 44%, 12% and 12% respectively when compared to the seeded α-syn formed in the absence of the compounds at 30D (Fig. 3F). This demonstrates that compounds D-520 and D-519 are much better inhibitors to α-syn aggregation and that they also lead to a substantially greater decrease in the toxicity of the aggregates formed in their presence when compared to their parent compounds or the reference compound Rifampicin.

TEM images showed a predominant presence of large fibrillar structures at the end of 30D with seeding (Fig. 5C) with relatively smaller sizes of aggregates at 10D (Fig. 5B). Further analysis by CD spectroscopy revealed the conversion from unordered structures towards a more structured form with an increase in the incubation time as expected (Fig. 6A). However, α-syn samples without seeding did not show any change in the CD spectra data (data not shown). Our analysis of morphologies of aggregates in presence of the drugs by TEM showed that both D-520 and D-519 were able to reduce the aggregation of α-syn and lead to the formation of small amorphous aggregates along with very few fibrils (Fig. 5D–G). In this regard, as shown in Fig. 5D–G and also in the supplemental section (Supplemental Figure 4A–C) the compounds themselves do not form any aggregates. The CD spectra analysis revealed that D-520 and D-519 lead to a decrease in the spectra at 202 nm and an increase in the spectral reading at 222 nm. However, this value was in between that of the unseeded and seeded samples showing that the degree of aggregation has been brought down by our compounds (Fig. 6B). This shows that our compounds are capable of binding to α-syn aggregates, thereby preventing them from further seeding.

It has been shown that expression of wild type and disease-related, mutated variants of α-syn in *Drosophila* neurons leads to degeneration^55^. We next evaluated compounds D-520 and D-519 in α-syn dependent toxicity in a model of synucleinopathy in *Drosophila* (Figs 7 and 8). The fruit fly model was first validated with the positive reference control drug Rifampicin, which suppressed α-syn dependent toxicity in the fly eyes compared to vehicle treated flies (Fig.7A,B). The fact that flies were fed Rifampicin only as adults, not throughout their development, is a testament to the power of this technique and to the efficacy of Rifampicin to suppress α-syn dependent degeneration in *Drosophila*. We were then able to demonstrate that both D-519 and D-520 rescued fly eyes from toxicity of α-syn over a period of time, from day 7 through 28 (Fig. 8A,B). We also found a concentration dependent protection by D-520, with strongest protection observed with 5 mg/mL concentration (Fig. 8C,D) on day 7. Thus, our results from fly experiments correlate with the data obtained from our new *in vitro* assay.

In brief, we have been able to demonstrate the development of a novel *in vitro* assay to screen possible PD therapeutics and to evaluate their effects in inhibiting aggregation of α-syn and toxicity of aggregates. Furthermore, through the utilization of our assay we have been able to demonstrate modulatory properties of α-syn aggregation and toxicity by our novel multifunctional agonists D-520 and D-519. Our results were further validated in an *in vivo* α-syn dependent toxicity model of synucleinopathy in *Drosophila*. In the development of our assay, we utilized a novel approach which involved seeding of α-syn with a low concentration of PFFs to initiate the aggregation process by recruiting the monomers to form aggregates. Our approach mimics the recent findings from *in vivo* animal model study involving intrastriatal administration of PFF in mice and rats resulting in an increased α-syn pathology accompanying the formation of LB type inclusions^24^,^45^. Importantly, our approach is quite different from other assays as we do not apply any external forces to induce the aggregation process. Interestingly, we have demonstrated that unlike the multifunctional dopamine agonists D-519 and D-520 developed by us, known dopamine agonists Pramipexole and 5-OHDPAT were much less effective in this assay. The results from our assay were further validated in an *in vivo Drosophila* synucleinopathy model. The significant protection of fly eyes from toxicity of α-syn by both compounds corresponded well with the results from *in vitro* assay. Thus, this new assay might offer a more reliable *in vitro* screening of α-syn inhibitors before advancing to *in vivo* experiments. Our future studies with more test compounds should further validate the assay.

## Materials and Methods

### Reagents

Rifamipicin, Thioflavin T, Thiazolyl Blue Tetrazolium Bromide (MTT) were purchased from Sigma Aldrich. Uranyl acetate was purchased from Electron microscopy sciences. Dimethyl sulfoxide (DMSO) and methanol were purchased from Fischer Scientific.

### Cell Culture

PC12 Adh (ATCC CRL1721.1) cells, a rat adrenal pheochromocytoma cell line, were purchased from ATCC. RPMI 1640, heat-inactivated horse serum, fetal bovine serum, penicillin-streptomycin, and trypsin were purchased from Gibco. PC12 cells were culture in RPMI 1640 medium supplemented with 10% heat-inactivated horse serum, 5% fetal bovine serum, and 1% Penicillin Streptomycin (100X, 10,000 Units/ml penicillin, 10,000 g/ml streptomycin) at 37 °C in 5% CO_2_ atmosphere. PC12 cells between the passage numbers 5–15 were used for the experiments.

### Purification of α-syn

The plasmid pET28 containing α-syn was a generous gift from Dr. Chad M. Rienstra from University of Illinois. α-Syn purification was carried out according to Huang *et al*.^56^. α-Syn was expressed in Escherichia coli BL21(DE3) cells. A single colony was transferred to 50 ml of LB medium containing Kanamycin (30 μg/mL) and incubated at 37 °C overnight. This culture was diluted 100 fold and induced with 100 μM IPTG for a period of 5 h at 37 °C. Osmotic shock was carried out according to Shevchik *et al*.^57^. Briefly, the cell pellet from a 1 L culture was resuspended in 100 mL osmotic shock buffer (30 mM Tris–HCl, 40% sucrose, and 2 mM ethylene diamine tetra acetic acid disodium, pH 7.2) and incubated at room temperature for 10 min. The pellet collected by centrifugation at 12,000 rpm for 20 min was resuspended quickly with 90 mL cold water followed by the addition of 37.5 μL of saturated MgCl_2_, and kept on ice for 3 min. The supernatant containing periplasm proteins was collected by centrifugation at 12,000 rpm for 20 min, and dialyzed against buffer A (20 mM Tris–HCl, pH 8.0) overnight. After centrifugation at 12,000 rpm for 20 min, the supernatant was loaded onto a Hiload 26/10 Q-Sepharose High performance column (GE healthcare) and eluted with a 0–0.5 M NaCl gradient in buffer A. The elution fractions were analyzed by 15% SDS-PAGE, and the fractions containing only 18 kDa band were combined for dialysis against 20 mM NH_4_HCO_3_ and further lyophilized. Centrifugation, chromatography, and dialysis were all carried out at 4 °C.

### Preparation of α-syn fibrils

Lyophilized α-syn was dissolved in sterile PBS and filtered through a 0.2 μm filter to remove any pre-formed aggregates. α-Syn fibrils were generated by incubating purified α-syn at 5 mg/mL at 37 °C under constant agitation at 1000 rpm in a Thermomix R shaker (Eppendorf, Hamburg, Germany) for a period of 5 days. The fibrils generated were then aliquoted and stored at −80 °C until use^23^.

### Thioflavin T assay

The formation of the fibrils was confirmed by Thioflavin T assay. A volume of 42 μL of 500 mM ThT (1.1 mg of ThT in 7 mL of phosphate-buffered saline (PBS)) solution was mixed with 479 μL of PBS to obtain 40 μM ThT solution. Then 10 μL of protein was mixed with 10 μL of 40 μM ThT in a black 384-well plate (solid bottom, Corning), and the fluorescence was measured using the Synergy Hybrid H1 fluorescence microplate reader (BioTek) at 440 nm excitation and 485 nm emission wavelength with auto sensitivity mode.

### Seeding of α-syn monomers with pre-formed fibrils

All the samples were prepared in PBS. Lyophilized α-syn was dissolved in sterile PBS and was filtered through a 0.2 μm filter to remove any pre-formed aggregates. α-Syn at 1.25 mg/mL (86.45 μM) was seeded with either 1% or 0.5% v/v of PFFs at 37 °C without agitation either alone or in the presence of the compounds at twice the molar concentration (172.9 μM) of monomeric α-Syn. Samples were collected at, 10D, 20D and 30D time points. The formation of fibrils was confirmed by ThT assay. (Please refer to supplementary file for a detailed protocol).

### Evaluation of cytotoxicity of extracellular α-syn aggregates formed by seeding in cell culture system

PC12 cells were seeded at 17 000 cells/well density in a 96-well plate. 24 h following plating, the cells were treated with α-syn aggregates such that its concentration is 10 μM. 24 h following treatments, viability was measured by MTT assay. MTT at 5 mg/mL was added to the cells such that its volume was 1/10^th^ the volume of the media. Cells were incubated with MTT for a period of 3 h, following which the formazan crystals were solubilized in 100 μL of 1:1 mixture of DMSO/Methanol and the absorbance was measured at 570 and 690 nm using an Epoch microplate reader (BioTek, Winooski, VT). Background-corrected values (570−690 nm) were used to plot the graph. Data from at least three experiments were analyzed using GraphPad software (version 6, San Diego, CA).

### Transmission Electron Microscopy

A 4 μL aliquot of α-syn seeding samples was adsorbed onto a Formvar-coated, carbon-stabilized copper grid (400 mesh) for 4 min. The grid was then rinsed briefly with distilled water twice, negatively stained with 2% aqueous uranyl acetate, air-dried, and examined with a JEOL (JEM 2010) transmission electron microscope at an accelerating voltage of 200 kV and 80,000 or 120,000 magnification.

### CD measurements

CD spectra were obtained with a Jasco J-1500 CD spectrophotometer. α-Syn samples which were prepared in PBS were diluted to 20 μM in ultrapure water for CD spectra measurements. Spectra were recorded in a 0.01-cm cell from 260 to 190 nm with a step size of 1 nm and a bandwidth of 1 nm. For all spectra, an average of five scans was obtained. CD spectra of the appropriate buffer was recorded and subtracted from the protein spectra.

### *Drosophila* studies

*Drosophila* stocks were raised and maintained in standard cornmeal media at 25 °C and ~40–60% humidity with 12 h diurnal cycle. For experiments, flies were crossed, raised and maintained at 30 °C in standard media, diurnal cycle and at ~40–60% humidity. Once offspring eclosed from their pupal cases (Day 0), they were switched (on Day 1) to custom-made instant fly food (Genesee Scientific) containing either D-519 or D-520 at a final concentration noted in figures and legends (vehicle was ultra pure water) or Rifampicin (Sigma-Aldrich) at 1 mg/mL (vehicle was DMSO) and maintained at 30 °C. Vehicle control flies were maintained in the same type of food, and at the same conditions.

At specific time points, fly heads were dissected for fluorescence imaging. Dissected heads were imaged using an Olympus BX53 microscope equipped with a DP72 digital camera, and fluorescence from fly eyes was quantified as previously described^48^,^49^. All flies in each experimental setup were examined and imaged using the same conditions. UAS-α-syn fly stock was number 8147 from Bloomington *Drosophila* Stock center. mCD-GFP fly stock was number 892 from Bloomington *Drosophila* Stock center.

### Statistical analysis

Statistical analyses were performed using GraphPad Prism Version 6 (GraphPad Software, San Diego, CA, USA). We analyzed all the data by One way analysis of variance (ANOVA) followed by Tukey’s multiple comparison post hoc test.

## Additional Information

**How to cite this article**: Yedlapudi, D. *et al*. Inhibition of alpha-synuclein aggregation by multifunctional dopamine agonists assessed by a novel *in vitro* assay and an *in vivo Drosophila* synucleinopathy model. *Sci. Rep.* **6**, 38510; doi: 10.1038/srep38510 (2016).

**Publisher's note:** Springer Nature remains neutral with regard to jurisdictional claims in published maps and institutional affiliations.

## Supplementary Material

Supplementary Information

## Figures and Tables

**Figure 1:**
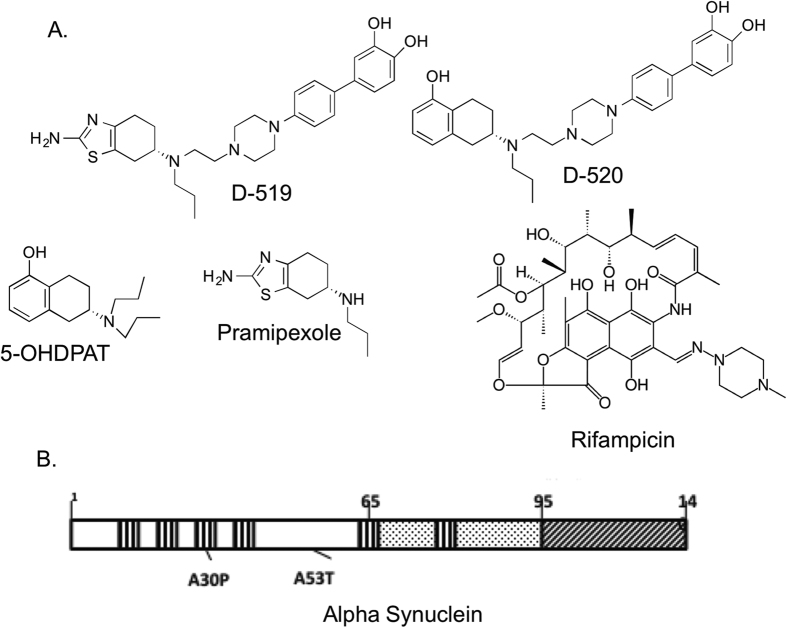
(**A)** Molecular structures of D-519, D-520, Rifampicin, 5-Hydroxy DPAT and Pramipexole. (**B**) α-Syn structure and domains: The N terminus domain is shown in white, the non amyloid component domain in dotted region and the C terminus domain in diagonal lines. The KTKEGV imperfect repeats are shown in vertical lines.
