# Supplementary material for: Inhibition of alpha-synuclein aggregation by multifunctional dopamine agonists assessed by a novel in vitro assay and an in vivo Drosophila synucleinopathy model
Source: Sci Rep. 2016 Dec 5;6:38510. doi: 10.1038/srep38510 (PMC5137034; doi:10.1038/srep38510)
Supplement: Supplementary Information [file srep38510-s1.pdf]

Supplementary Materials

Inhibition of alpha-synuclein aggregation by multifunctional dopamine agonists assessed by a novel *in vitro* assay and an *in vivo Drosophila* synucleinopathy model

Deepthi Yedlapudi[1], Gnanada S. Joshi[2], Dan Luo[1], Sokol V. Todi[2], Aloke K. Dutta[1*]

From the [1]Department of Pharmaceutical Sciences, Wayne State University, Detroit, MI 48202, [2]Department of Pharmacology, Wayne State University, Detroit, MI 48202.

Corresponding author:

Aloke K. Dutta, Ph.D., Department of Pharmaceutical Sciences, Eugene Applebaum College of Pharmacy & Health Sciences, Wayne State University, Detroit, MI 48202

Tel: 1-313-577-1064, Fax: 1-313-577-2033, e-mail: adutta@wayne.edu

Supplementary file 1:

Materials and methods: Seeding of α-syn monomers with preformed fibrils

In order to make monomeric α-syn at a concentration of 86.45 µM, we initially make a stock of 172.9 µM. Equal volumes of 172.9 µM stock and PBS were mixed to get the 86.45 µM monomeric α-syn solution.

For making, 1% seeding sample, initially a stock solution of α-syn at a concentration of 172.9 µM with 2% PFFs is made. Equal volumes of this solution are mixed with PBS to get 86.45 µM with 1% PFFs. In a similar way, for making 0.5% seeding sample, initially a stock solution of α-syn at a concentration of 172.9 µM with 1% PFFs is made. Equal volumes of this solution are mixed with PBS to get 86.45 µM with 0.5% PFFs.

In order to make 0.5% seeding samples with the compounds, equal volumes of α-syn at a concentration of 172.9 µM with 1% PFFs are mixed with 345.8 µM compound solution. This will result in a solution with 86.45 µM with 0.5% PFFs and 172.9 µM compounds.

Supplementary Fig. 1: Preparation of α-syn fibrils:

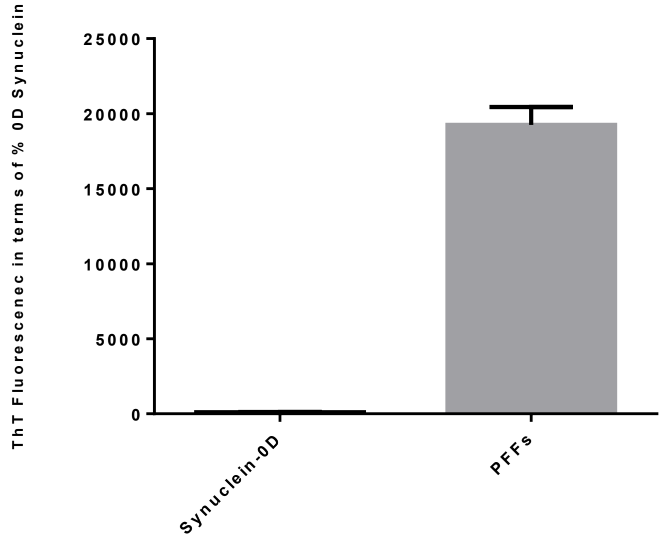

Supplementary Fig. 1: 5mg/mL α-syn was incubated at 37ºC with constant agitation at 1000rpm for a period of 5days. The degree of fibrillation of α-syn was measured by ThT assay.Values are represented in terms of % 0D Synuclein. Data values shown are means ± SD of two experiments.

Supplementary Fig. 2: Effect of 1% PFF on the aggregation of monomeric α-syn:

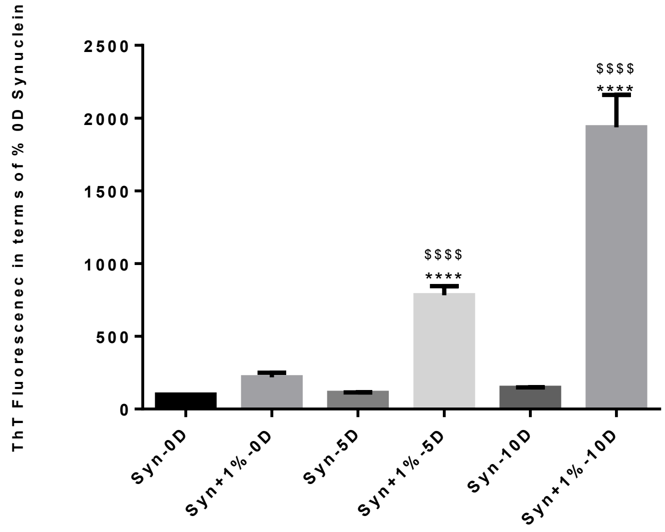

Supplementary Fig. 2: 1.25 mg/mL α-syn was incubated with 1% PFFs for a period of 10D and fibrillation was measured by ThT assay. Values are represented in terms of % 0D Synuclein. Data values shown are means ± SD of three independent experiments. One-way ANOVA analysis followed by Tukey's multiple comparison post hoc test was performed. (****$p \leq 0.0001$ compared to Syn-0D, $$$$$p \leq 0.0001$ compared to the Syn+1%-0D)

Supplementary file 2:

Native gels, western blotting and quantification: To obtain mechanistic clues into the manner in which select drugs that showed protection in *Drosophila* reduced toxicity from α-syn, we examined monomeric and aggregated species of the toxic protein in dissected fly eyes as described below. Post-fluorescence imaging, dissected fly heads were homogenized for use with the NativePAGE™ Novex® Bis-Tris Gel System (Life Technologies), per the manufacturer's recommendations. Fly heads were mechnaically disrupted in 4X Native sample buffer. Lysates were centrifuged at 20,000 × g for 30 minutes at 4 °C, then the supernatant was loaded onto gradient 3-12% pre-cast Native gels (BN1003BOX, Life Technologies) and proteins were electrophoresed in 1X light blue Cathode buffer containing 0.5% NativePAGE™ Cathode Additive (BN2002, Life Technologies). Proteins were transferred onto a PVDF membrane and detected using anti-α-syn antibody (SC-7011-R, Santa Cruz, Biotechnology; 1:500). Anti-tubulin antibody (Sigma Aldrich; 1:5000) was used as loading control. Peroxidase conjugated secondary antibodies: goat anti-rabbit and goat anti-mouse (Jackson Immunoresearch; 1:5000) were used for visualization using chemiluminiscence (Clarity TM Western ECL Substrate, Bio-Rad) on a CCD-equipped VersaDoc 5000MP system (Bio-Rad). The signals from the

western blot were quantified using the Quantity One Software (Bio-Rad) with global noise reduction and using non-saturated blots as described before [1-4].

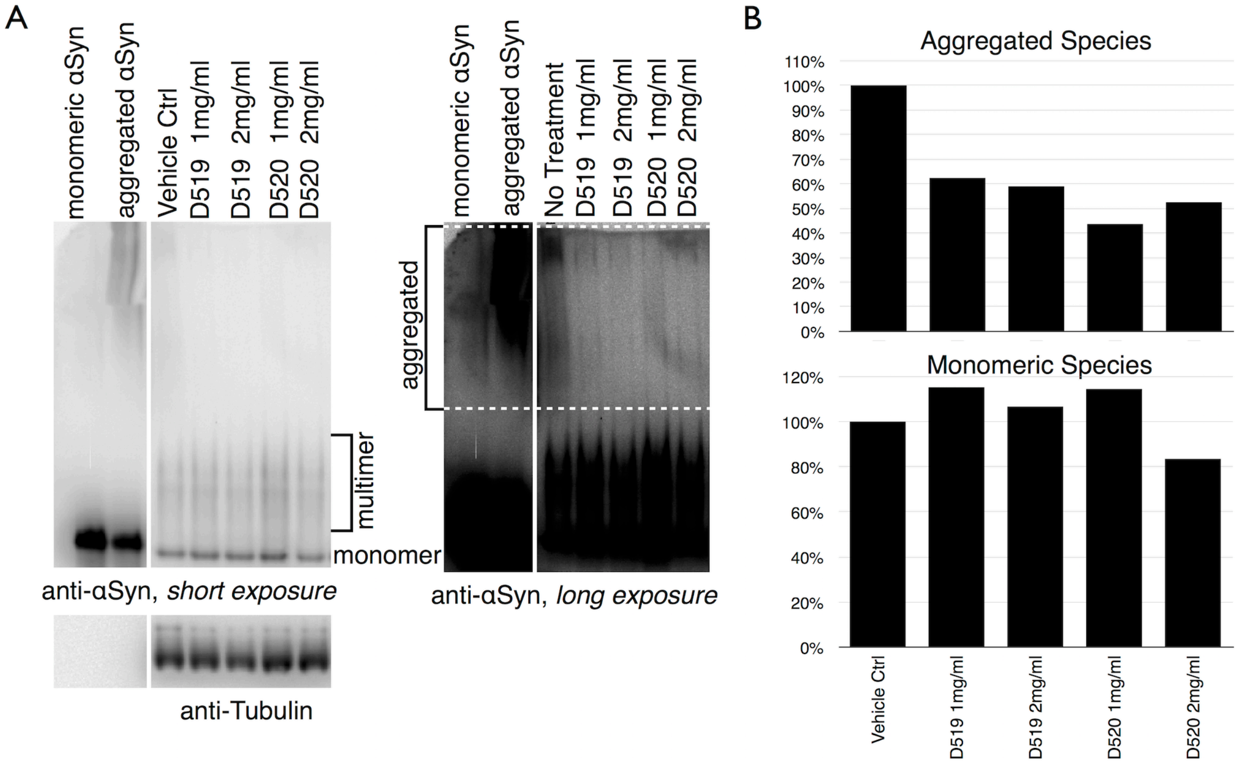

Supplementary Figure 3: A) Native PAGE western blots from recombinant α-syn (monomer and aggregated, left portion) or from dissected fly heads expressing α-syn and treated as indicated in the right portion of the gel. 10 dissected fly heads were used per group. B) Quantification of the data from panel A, where monomeric and aggregated α-syn species are normalized to the Vehicle treatment control. Flies were 28 days old. For native gels, the NativePAGE™ Novex® Bis-Tris Gel System (Life Technologies) was used, following the recommendations of the manufacturer. Lanes are from the same blot and exposures, cropped and rearranged for ease of viewing.

As shown in Fig 3, the larger aggregated species of α-syn were markedly reduced by D-519 and D-520, compared to the vehicle treated controls. Monomeric α-syn did not seem to be affected much. Together with the GFP-based assays, these results are consistent with a model that D-519 and D-520 suppress α-syn toxicity by reducing its aggregative propensity *in vivo* in the fruit fly.

Transmission Electron Microscopy Study

Further TEM study was carried out to observe whether any aggregates are formed by the compounds themselves. As shown in Figure 4, the TEM analysis of solution of compounds D-519 and D-520 at the same concentration as used in the inhibition study (172.9 μM) shows no formation of aggregates by the compounds alone.

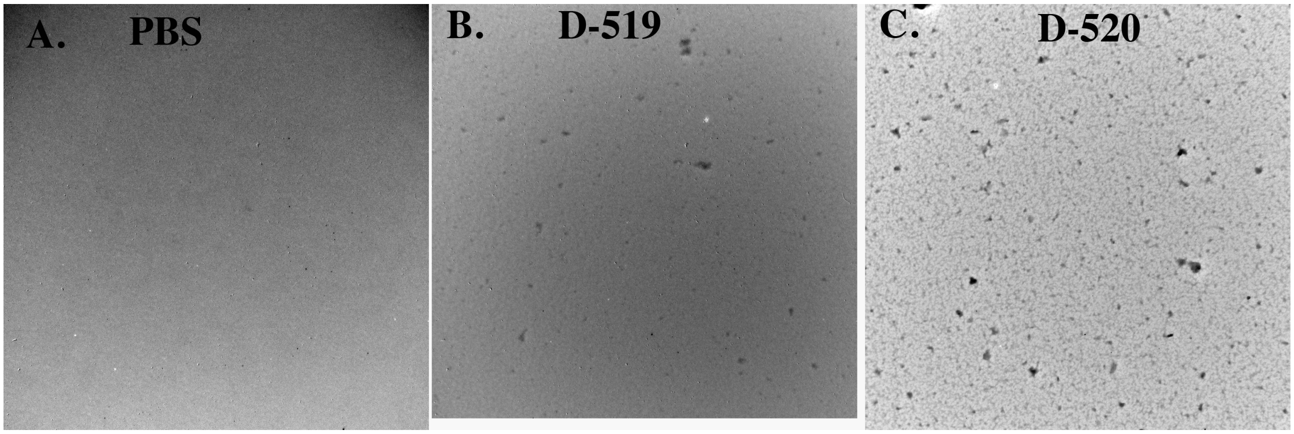

Figure 4: TEM analysis of solution of A) PBS, B) 172.9 μM D-519 and C) 172.9 μM D-520.

References:

1 Winborn, B. J. *et al.* The deubiquitinating enzyme ataxin-3, a polyglutamine disease protein, edits Lys63 linkages in mixed linkage ubiquitin chains. *The Journal of biological chemistry* **283**, 26436-26443, (2008).

2 Blount, J. R. *et al.* Ubiquitin-binding site 2 of ataxin-3 prevents its proteasomal degradation by interacting with Rad23. *Nature communications* **5**, 4638, (2014).

3 Blount, J. R., Burr, A. A., Denuc, A., Marfany, G. & Todi, S. V. Ubiquitin-specific protease 25 functions in Endoplasmic Reticulum-associated degradation. *PloS one* **7**, e36542, (2012).

4 Tsou, W. L. *et al.* The deubiquitinase ataxin-3 requires Rad23 and DnaJ-1 for its neuroprotective role in Drosophila melanogaster. *Neurobiology of disease* **82**, 12-21, doi:10.1016/j.nbd.2015.05.010 (2015).
